# Quality of life is associated with physical activity and fitness in cystic fibrosis

**DOI:** 10.1186/1471-2466-14-26

**Published:** 2014-02-27

**Authors:** Helge Hebestreit, Kerstin Schmid, Stephanie Kieser, Sibylle Junge, Manfred Ballmann, Kristina Roth, Alexandra Hebestreit, Thomas Schenk, Christian Schindler, Hans-Georg Posselt, Susi Kriemler

**Affiliations:** 1 Pediatric Department, University of Würzburg, Würzburg, Germany; 2 Pediatric Pulmonology and Neonatology, Hannover Medical School, Hannover, Germany; 3 St Josef Hospital Pediatric Clinic, Ruhr University Bochum, Bochum, Germany; 4 Department of Anaesthesiology, University of Würzburg, Würzburg, Germany; 5 Swiss Tropical and Public Health Institute, University of Basel, Basel, Switzerland; 6 Pediatric Department, Johann Wolfgang Goethe University, Frankfurt, Germany; 7 Institute for Social and Preventive Medicine, University of Zürich, Zürich, Switzerland; 8 Universitäts-Kinderklinik, Josef-Schneider-Str. 2, 97080 Würzburg, Germany

**Keywords:** Exercise testing, Oxygen uptake, Longitudinal analysis, Accelerometry, Questionnaire

## Abstract

**Background:**

Health-related and disease-specific quality of life (HRQoL) has been increasingly valued as relevant clinical parameter in cystic fibrosis (CF) clinical care and clinical trials. HRQoL measures should assess – among other domains – daily functioning from a patient’s perspective. However, validation studies for the most frequently used HRQoL questionnaire in CF, the Cystic Fibrosis Questionnaire (CFQ), have not included measures of physical activity or fitness. The objective of this study was, therefore, to determine the cross-sectional and longitudinal relationships between HRQoL, physical activity and fitness in patients with CF.

**Methods:**

Baseline (n = 76) and 6-month follow-up data (n = 70) from patients with CF (age ≥12 years, FEV1 ≥35%) were analysed. Patients participated in two multi-centre exercise intervention studies with identical assessment methodology. Outcome variables included HRQoL (German revised multi-dimensional disease-specific CFQ (CFQ-R)), body composition, pulmonary function, physical activity, short-term muscle power, and aerobic fitness by peak oxygen uptake and aerobic power.

**Results:**

Peak oxygen uptake was positively related to 7 of 13 HRQoL scales cross-sectionally (r = 0.30-0.46). Muscle power (r = 0.25-0.32) and peak aerobic power (r = 0.24-0.35) were positively related to 4 scales each, and reported physical activity to 1 scale (r = 0.29). Changes in HRQoL-scores were directly and significantly related to changes in reported activity (r = 0.35-0.39), peak aerobic power (r = 0.31-0.34), and peak oxygen uptake (r = 0.26-0.37) in 3 scales each. Established associates of HRQoL such as FEV1 or body mass index correlated positively with fewer scales (all 0.24 < r < 0.55).

**Conclusions:**

HRQoL was associated with physical fitness, especially aerobic fitness, and to a lesser extent with reported physical activity. These findings underline the importance of physical fitness for HRQoL in CF and provide an additional rationale for exercise testing in this population.

**Trial registration:**

ClinicalTrials.gov, NCT00231686

## Background

In cystic fibrosis (CF) patient care and in CF-related clinical trials, patient reported outcomes such as health-related quality of life (HRQoL) have been increasingly valued as relevant clinical parameter over the last two decades [1,2]. HRQoL even allowed a prediction of survival in patients with CF [3].

HRQoL measures should assess – among other domains – daily functioning from a patient’s perspective [4,5]. Thus, one would expect a direct association of HRQoL with physical activity and physical fitness. In healthy people, a positive relationship has consistently been found between physical activity [6] or aerobic fitness [7] and HRQoL, indicating their significant contribution to perceived well-being. Research is scarce which has been done to explore the importance of their role in people with chronic conditions such as CF. The few existing studies showed moderate to strong direct correlations between HRQoL based on the non disease-specific Quality of Well-being Scale and objectively measured physical activity [8] or peak oxygen uptake [9]. However, the use of generic HRQoL questionnaires in CF may be questioned as they do not include disease-specific aspects [4,10].

The revised Cystic Fibrosis Questionnaire (CFQ-R) is a validated, multi-dimensional disease-specific measure to assess generic and disease-specific domains of HRQoL in CF that is available in several languages including English and German [5,11]. To our knowledge, only one relatively small cross-sectional study has assessed the association between HRQoL-scales and aerobic fitness so far [12] and found a positive relationship between VO_2_peak and several scales, which did not persist after adjusting for age and gender [12].

Based on the scarce cross-sectional [12] and completely lacking longitudinal data in CF, the objective of this study was to determine the cross-sectional and longitudinal relationships between HRQoL as assessed by the CFQ-R, and physical activity and fitness in this population. Based on the studies using the Quality of Well-being Scale we hypothesised that there would be significant and positive associations between HRQoL scales and measures of physical activity and physical fitness cross-sectionally and longitudinally.

## Methods

### Study population

Data for this project were taken from two exercise intervention studies in CF that were designed as twin studies with different objectives but identical assessment methodology: a German study with a partially supervised training group and a control group, and a Swiss study with a supervised aerobic training group, a supervised strength training group and a control group [13,14]. Patients with CF, aged 12 years and older and with an FEV1 of at least 35% of predicted, were recruited from German CF centers in Frankfurt, Hannover, and Würzburg (n = 38), and from the German-speaking provinces of Switzerland (n = 39). The respective ethics committees of the participating centers (Frankfurt, Hannover, Würzburg for Germany and Zürich for Switzerland) approved the study protocol and procedures. Written informed consent was obtained from the patients and their guardians, if the patients were under 18 years of age.

At the end of the baseline assessments, patients were randomly allocated to an intervention (n = 23) or a control group (n = 15) in Germany and to a strength-training (n = 12), an aerobic training (n = 17) and a control (n = 10) group in Switzerland by drawing lots out of an opaque bag which contained 24 lots intervention and 16 lots control in Germany, and 20 lots each for the aerobic training, strength training and the control group in Switzerland. The initial goal of recruiting 60 Swiss CF patients could not be reached. One male 16-year-old Swiss patient did not complete the quality of life questionnaire at baseline so that his data were also not included in this analysis. Until the follow-up visit after 6 months, three patients from Germany (all from the intervention group) and three patients from Switzerland (two from the aerobic training group, and one from the strength-training group) decided to discontinue their participation in the study.

### Study design

Patients were seen in their respective centre at study entry (Germany: December 2000 to March 2001; Switzerland: September 1999 to June 2001), and after 6 months. All measurements were taken while participants were in stable clinical condition. If needed, visits were postponed until the clinical status was stable or intravenous treatment was over for at least 4 weeks. At each visit, the following outcome variables were determined: HRQoL, reported physical activity, height, weight, and body mass index, body composition (percent body fat), pulmonary function (forced expiratory volume in 1 s – FEV1), short-term muscle power, maximal aerobic work rate, and peak oxygen uptake (VO_2_peak). Within a period of ± 3 weeks around the above visits, time spent in moderate-and-vigorous physical activity (MVPA) was determined by accelerometry.

### Procedures

At baseline and at 6-months follow-up, HRQoL was assessed by the adolescent and adult versions of the revised German CFQ-R questionnaire [11]. This self-administered, disease-specific health-related questionnaire has five generic scales (Physical functioning, Vitality, Emotional state, Social limitations, Role limitations), four disease-specific scales (Feelings of embarrassment about symptoms, Eating disturbance, Body image, Treatment burden), one scale on Subjective general health perception, and three scales assessing Respiratory symptoms, Digestive symptoms and Weight problems. For each scale, the score is given on a 0- to 100-point scale with higher scores denoting higher HRQoL, i.e. better functioning or health perception and less problems or symptoms.

The German CFQ-R [11] includes a scale on “Embarrassment’ which has been omitted from the United States CFQ-R [5]. Reported scale consistencies of the German CFQ-R are satisfactory (Cronbach α > 0.7) for all scales except for the ‘Digestive symptom’ scale (α = 0.66) [11]. Test-retest reliability was high for all HRQoL-scales in the previous version of that instrument (intraclass correlation coefficients 0.76-0.92) [11]. Construct validity was shown by significant correlation coefficients between most CFQ-R scales and FEV1 and for some scales with BMI [11]. Significant improvements in most CFQ-R scales following a rehabilitation program have demonstrated responsiveness of the German CFQ-R [11]. In general, validation studies for the German and US CFQ-R have shown similar reliability, validity and responsiveness [5,11].

BMI was calculated from height and body mass, and body adiposity from skinfold thickness that was converted into percent body fat (% BF) [15,16]. FEV_1_ and FVC were determined by standard spirometry and expressed as % predicted [17]. Short-term muscle power was assessed on a calibrated mechanically braked cycle ergometer using the 30-sec Wingate test protocol [18] (in Germany: Monark 834 E Ergomedic ergometer (Varberg, Sweden); in Switzerland: Fleisch cycle ergometer (Metabo, Switzerland)). There is good agreement among ergometers albeit tested in young children [19]. For each Wingate test, braking force was calculated from body mass using existing equations [18] and modified up to 10% depending on the performance in two short practice runs. Average power generated during the Wingate test was chosen as indicator of muscle power and expressed relative to body weight.

After a rest period of at least 30 min, subjects completed a continuous incremental cycling task to volitional fatigue following the protocol proposed by Godfrey et al. [20]. Work rate was increased every minute by 15 to 20 Watts, depending on patient´s height and physical fitness. German patients were tested using a calibrated Monark 834 E cycle ergometer and metabolic cart (CPX/D, MedGraphics, St Paul, MN) while Swiss patients used a Fleisch cycle ergometer and a Quark metabolic cart (Cosmed, Rome, Italy) The metabolic carts were calibrated before each exercise test with two gases of known concentrations. Stability of the O_2_- and CO_2_-sensors was verified after each test. Maximal aerobic power (Wmax) was taken as the power maintained over the final completed 1-minute stage during the test. Peak oxygen uptake (VO_2_peak) was determined as highest VO_2_ over 30 s during the test and expressed relative to body weight. Wmax and VO_2_peak were also expressed in % predicted [20,21]. The values expressed in % predicted were used for further analyses.

Total time per week reported in the categories ‘hard’ and ‘very hard’ of the 7-day Physical Activity Recall Questionnaire [22,23], translated into German was used as measure of reported activity. This questionnaire has recently been validated for patients with CF [24].

Within ± 3 weeks to the baseline and follow-up assessments, patients wore a MTI/CSA 7164 accelerometer (MTI Health Services, Fort Walton Beach, FL) for 7 consecutive days at the right hip with an epoch time set to 60 s. Periods of 60 min or more with zero readings were excluded from analysis. Data were included if a patient had completed at least 5 days of recordings with at least 10 hours of valid data per day. Time spent in moderate and vigorous physical activity (MVPA) was defined based on cut-offs above 1000 counts · min^-1^, respectively, and expressed in hours per week [13,25].

Details of the interventions are provided in the respective publications [13,14]. Briefly, patients of the intervention groups consented to add regular strenuous exercise to their baseline activities for 6 months. In the Swiss study, weekly exercise was increased by three times 30 min of supervised strength training in a fitness centre (whole body strengthening with one set of 15 repetitions) or aerobic training (on a stationary cycle ergometer at 65% of maximal oxygen uptake) in a fitness centre or at home. Supervision of the training was done by the same persons throughout the study; in the aerobic training group by a student involved in the study, in the strength training group by staff of the respective fitness centers, in the German study with regular phone calls by one investigator (StKi). In the German study, patients of the intervention group agreed to add three times 60 min per week of any sport activity to their routine activities. They could freely choose the type of activity for each exercise session.

### Analyses

Data are described as means ± SD or median ± interquartile range (IQR). HRQoL data are presented in both versions due to comparability to other studies in this area. Non-parametric tests were used to assess differences among groups and effects of potential predictors on the ordinal and discontinuous HRQoL scales. Differences in baseline characteristics according to gender and country were compared by rank sum test. Associations between HRQoL scales and anthropometry, lung function, physical activity and fitness were tested by Spearman rank correlation analyses. Likewise, Spearman rank correlation analyses were used to determine the relationships between the changes on HRQoL scales over 6 months and the changes in the above predictors over this time period. We also tested associations between HRQoL-scales and BMI, body fat, FEV1, physical activity, and anaerobic and aerobic performance at baseline and changes over 6 months by linear regression models with adjustments for age, gender, group allocation (training vs no training) and nationality. Since HRQoL scores are ordinal variables with a few discrete stages, bootstrapping was used to estimate standard errors of the regression-coefficients. We finally used the simple correlation models as results were similar when using simple correlation analyses or multiple linear regression models with bootstrapping adjusting for best and most plausible set of predictors (age, gender, and nationality for the cross-sectional analyses; age, gender, nationality, training vs no training, strength training vs other modalities of training for the longitudinal analyses with bootstrapping (see Additional file 1). Statistical significance was accepted at p < 0.05.

## Results

Data of 76 patients were available for cross-sectional analyses while 70 patients contributed data to longitudinal analyses covering 6 months (Table 1).

**Table 1:** Number of patients with complete data at baseline and 6 months for each indicated measurement

	**Baseline**	**6-month follow-up**	**Both**
Body mass index (kg/m^2^)	76	70	70
FEV1 (% predicted)	76	70	70
Reported activity (hours per week)	71	68	64
Measured MVPA (hours/week)	70	54	53
Mucle power (W/kg)	74	70	68
Wmax (% predicted)	76	69	69
VO_2 _peak (% predicted)	73	69	66
Quality of life*	76	70	70

FEV1 – forced expiratory volume in 1 second; MVPA – moderate-and-vigorous physical activity; Wmax – maximal aerobic power; VO2peak – peak oxygen uptake. *for the scale role limitations only 66 patients provided data at baseline, 59 at the 6-months follow-up and 52 had complete data at both time points to calculate changes.

Tables 2 and 3 describe baseline characteristics and their respective changes over 6 months of the population. Male and female patients differed significantly in their baseline anthropometric variables for reported physical activity and muscle power. Swiss patients were significantly older than the German patients (21.6 ± 5.4 vs. 19.6 ± 6.0 years, p < 0.05) and had a lower aerobic power (77 ± 13 vs 84 ± 15% predicted, p < 0.05) and less body fat (13.9 ± 7.2 vs 17.5 ± 7.1%, p < 0.05). Except for the HRQoL scale ‘Role limitations’ which was significantly lower in females at baseline, there were no significant differences in HRQoL scales between genders, Swiss and German patients (data not shown), nor among the five intervention arms (data not shown) cross-sectionally nor over 6 months with one exception. There was a difference in changes in ‘Vitality’ among the five treatment groups which could be attributed to a significant decrease in ‘Vitality’ scores in the weight training group compared to the partially supervised training group (-8.3 (-16.7-0.0) vs. 0.0 (-8.3-8.3), p < 0.05).

**Table 2:** Subjects' characteristics at baseline and changes over 6 months according to gender

	**Baseline**			**Change over 6 months**		
	**Female**	**Male**	**Total**	**Female**	**Male**	**Total**
Number	37	39	76	34	36	70
Age (years)	19.5 ± 5.5	21.6 ± 5.9	20.6 ± 5.8	0.6 ± 0.1	0.6 ± 0.1	0.6 ± 0.1
Body mass index (kg/m^2^)	19.9 ± 2.5	19.8 ± 2.9	19.8 ± 2.7	–0.1 ± 1.2	0.1 ± 0.7	0.0 ± 1.0
FEV1 (% predicted)	72.3 ± 20.3	65.1 ± 17.4	68.6 ± 19.1	–1.3 ± 11.4	0.2 ± 11.3	–0.5 ± 11.3
Reported activity (hours/week)	5.8 ± 6.4	9.5 ± 10.6*	7.7 ± 9.0	1.1 ± 7.4	–2.5 ± 11.0	–0.7 ± 9.5
Measured MVPA (hours/week)	9.2 ± 3.8	11.2 ± 5.1	10.1 ± 4.5	–0.5 ± 4.7	–1.2 ± 3.4	–0.8 ± 4.1
Muscle power (W/kg)	5.6 ± 0.7	7.3 ± 1.1***	6.5 ± 1.3	–0.1 ± 0.8	–0.2 ± 0.7	–0.2 ± 0.7
Wmax (W)	152 ± 28	191 ± 44	172 ± 42	3 ± 23	14 ± 28	9 ± 26
Wmax (% predicted)	102.7 ± 19.3	94.7 ± 17.1	98.6 ± 18.5	1.0 ± 015.7	5.4 ± 13.6	3.3 ± 14.7
VO_2_peak (ml/kg/min)	33.9 ± 6.6	41.0 ± 6.7	37.4 ± 7.5	0.3 ± 5.4	1.6 ± 5.1	0.9 ± 5.2
VO_2_peak (% predicted)	82.6 ± 16.3	78.0 ± 13.2	80.3 ± 14.9	0.5 ± 13.9	3.0 ± 10.8	1.8 ± 12.4

Data are mean ± standard deviation (SD). Abbreviations: *FEV1* forced expiratory volume in 1 second, *MVPA* moderate–and–vigorous physical activity, *Wmax* maximal aerobic power, *VO2peak* peak oxygen uptake. *, **, *** denote a significant difference between female and male patients at the p < 0.05, p < 0.01 and p < 0.001 level, respectively.

**Table 3:** Health-related quality of life (A. median ± IQR; B. means ± SD) at baseline and changes over 6 months according to gender

**A. Medians ± IQR**	**Baseline**			**Change over 6 months**		
	**Female**	**Male**	**Total**	**Female**	**Male**	**Total**
Physical functioning	87.5 (75.0–95.8)	91.7 (75.0–100.0)	87.5 (75.0–95.8)	–3.1 (–8.3–5.2)	0.0 (0.0–7.3)	0.0 (–5.2–5.2)
Vitality	58.3 (50.0–70.8)	66.7 (50.0–75.0)	58.3 (50.0–75.0)	0.0 (–8.3–8.3)	0.0 (–8.3–14.6)	0.0 (–8.3–8.3)
Emotional state	86.7 (70.0–93.3)	86.7 (80–93.3)	86.7 (73.3–93.3)	0.0 (–6.7–8.3)	0.0 (–6.7–5.0)	0.0 (–6.7–6.7)
Social limitations	83.3 (58.3–91.7)	75.0 (58.3–91.7)	75.0 (58.3–91.7)	0.0 (–12.5–12.5)	0.0 (–8.3–6.3)	0.0 (–8.3–8.3)
Role limitations	87.5 (75.0–100.0)	100.0 (87.5–100.0)*	100.0 (87.5–100.0)	0.0 (0.0–12.5)	0.0 (0.0–0.0)	0.0 (0.0–9.4)
Feelings of embarrassment	83.3 (66.7–100.0)	77.8 (66.7–100.0)	77.8 (66.7–100.0)	0.0 (–11.1–11.1)	0.0 (–8.3–11.1)	0.0 (–11.1–11.1)
Body image	77.8 (66.7–88.9)	77.8 (55.6–88.9)	77.8 (66.7–88.9)	0.0 (–2.8–11.1)	0.0 (–11.1–8.3)	0.0 (–11.1–11.1)
Eating disturbances	100.0 (83.3–100.0)	100.0 (66.7–100.0)	100.0 (66.7–100.0)	0.0 (0.0–0.0)	0.0 (0.0–0.0)	0.0 (0.0–0.0)
Treatment burden	66.7 (50.0–83.3)	66.7 (50.0–83.3)	66.7 (50.0–83.3)	0.0 (–8.3–16.7)	0.0 (–16.7–16.7)	0.0 (–16.7–16.7)
Health perception	63.6 (54.5–81.8)	72.7 (63.6–90.9)	72.7 (62.8–81.8)	0.0 (–9.1–9.1)	0.0 (–7.1–9.1)	0.0 (–9.1–9.1)
Weight problems	100.0 (66.7–100.0)	66.7 (58.3–100.0)	100.0 (66.7–100.0)	0.0 (–25.0–0.0)	0.0 (–33.0–0.0)	0.0 (–33.0–0.0)
Respiratory symptoms	72.2 (64.3–91.0)	66.7 (61.9–76.2)	71.4 (61.9–77.8)	0.0 (–14.3–4.8)	0.0 (–9.5–9.5)	0.0 (–9.5–7.9)
Digestive symptoms	83.3 (66.7–100.0)	83.3 (66.7–100.0)	83.3 (66.7–100.0)	0.0 (0.0–16.7)	0.0 (–8.3–0.0)	0.0 (0.0–16.7)
**B. Means ± SD**	**Baseline**			**Change over 6 months**		
	**Female**	**Male**	**Total**	**Female**	**Male**	**Total**
Physical functioning	82.9 ± 16.2	85.6 ± 15.1	84.3 ± 15.6	–1.3 ± 13.3	2.2 ± 9.0	0.5 ± 11.4
Vitality	59.9 ± 16.2	63.2 ± 15.1	61.6 ± 15.6	–0.2 ± 16.6	3.0 ± 15.3	1.4 ± 15.9
Emotional state	81.4 ± 14.8	84.4 ± 15.4	83.0 ± 15.1	1.4 ± 16.1	–1.4 ± 9.7	0.0 ± 13.2
Social limitations	76.4 ± 19.0	72.0 ± 23.2	74.1 ± 21.2	–1.0 ± 23.5	0.6 ± 11.6	–0.2 ± 18.2
Role limitations	89.5 ± 12.5	95.0 ± 9.7*	92.4 ± 11.4	2.2 ± 14.9	–2.2 ± 18.9	–0.2 ± 17.2
Feelings of embarressment	77.2 ± 23.9	76.1 ± 23.3	76.6 ± 23.4	2.9 ± 20.0	1.2 ± 18.2	2.0 ± 19.0
Body image	77.2 ± 18.8	72.9 ± 23.9	75.0 ± 21.5	1.9 ± 17.9	–1.7 ± 15.8	0.0 ± 16.8
Eating disturbances	89.4 ± 17.0	86.8 ± 21.4	88.0 ± 19.3	1.0 ± 12.5	–0.5 ± 18.9	0.2 ± 16.0
Treatment burden	63.0 ± 18.1	65.0 ± 17.0	64.0 ± 17.8	2.5 ± 19.1	0.7 ± 19.6	1.6 ± 19.2
Health perception	67.5 ± 18.1	70.4 ± 19.5	69.0 ± 18.8	1.4 ± 19.3	0.7 ± 15.4	1.0 ± 17.3
Weight problems	83.8 ± 21.7	71.9 ± 32.4	77.8 ± 28.1	–8.3 ± 35.9	–7.1 ± 33.1	–7.7 ± 34.2
Respiratory symptoms	69.3 ± 15.8	67.6 ± 10.3	68.5 ± 13.3	–3.1 ± 19.0	–0.9 ± 10.0	–2.0 ± 15.1
Digestive symptoms	83.8 ± 14.4	83.3 ± 18.2	83.6 ± 16.3	4.5 ± 18.3	–1.0 ± 15.0	1.8 ± 16.8

*Denotes a significant difference between female and male patients at the p < 0.05 level.

### Relationship between HRQoL and established covariates

Cross-sectional and longitudinal correlation analyses are shown in Table 4. At baseline, patient’s age was correlated with more ‘Respiratory symptoms’ (r = -0.32, p < 001). BMI was positively related to the HRQoL scale ‘Eating disturbance’ (i.e. less eating disturbance), ‘Body image’ and ‘Weight problems’ (i.e. less weight problems). Body fat was directly related to the ‘Body image’ and ‘Weight problems’ scales. FEV1 was directly associated with ‘Physical functioning’, ‘Health perception’ and ‘Respiratory symptoms’ scores, i.e. increasing FEV1 values were associated with increasing values of physical functioning, health perception and decreasing respiratory symptoms (Table 4A). Changes in BMI over 6 months were directly associated with changes in ‘Health perception’ and ‘Weight problems’ scales (Table 4B), while changes in FEV1 were directly related to changes in ‘Feelings of embarrassment’ scores. The strength of the associations were moderate and ranged from r = 0.24-0.55.

**Table 4:** Relationships of baseline (A) and changes over 6 months (B) between quality of life scales and BMI, FEV1, physical activity measures, muscle power, and aerobic capacity

**A. Baseline**								
	**BMI**	**Body fat**	**FEV1**	**Reported activity**	**Measured MVPA**	**Muscle power**	**Wmax**	**VO**_**2**_**peak**
Physical functioning	0.098	–0.08	0.264*	0.2	0.118	0.320**	0.237*	0.369**
Vitality	0.055	–0.043	–0.034	0.213	0.012	0.304**	0.145	0.201
Emotional state	0.014	–0.092	0.024	0.184	0.035	0.176	0.153	0.204
Social limitations	–0.033	0.082	–0.066	0.212	–0.182	–0.031	0.06	0.059
Role limitations	0.03	–0.207	0.176	0.292*	0.222	0.296*	0.195	0.315*
Feelings of embarrassment	0.065	0.148	0.161	0.155	0.021	–0.051	0.072	0.174
Body image	0.435***	0.306**	0.217	–0.029	0.068	–0.171	0.092	0.164
Eating disturbances	0.373**	0.117	0.141	–0.006	0.111	0.034	0.167	0.297*
Treatment burden	0.096	–0.027	0.046	0.066	–0.134	0.137	0.211	0.177
Health perception	0.18	–0.023	0.283*	0.187	0.163	0.13	0.284*	0.396***
Weight problems	0.491***	0.228*	0.147	–0.073	0.012	–0.08	0.350**	0.456***
Respiratory symptoms	0.027	0.114	0.302**	0.106	0.057	0.041	0.204	0.424***
Digestive symptoms	0.057	–0.074	0.096	0.099	0.183	0.245*	0.307**	0.376**
**B. Changes over 6 months**								
	**Δ BMI**	**Δ Body fat**	**Δ FEV1**	**Δ Reported activity**	**Δ Measured MVPA**	**Δ Muscle power**	**Δ Wmax**	**Δ VO**_**2**_**peak**
Δ Physical functioning	–0.013	0.02	0.026	0.044	–0.198	0.012	0.056	0.114
Δ Vitality*	0.189	0.16	0.094	0.227	–0.16	0.057	0.305*	0.176
Δ Emotional state	0.154	0.174	0.073	–0.027	–0.143	0.097	0.137	0.002
Δ Social limitations	–0.134	0.075	0.1015	–0.027	–0.11	0.051	0.13	0.243
Δ Role limitations	0.123	–0.078	–0.077	0.393**	0.124	0.192	0.307*	0.267
Δ Feelings of embarrassment	0.109	0.035	0.244*	0.389**	0.037	–0.025	0.219	0.255*
Δ Body image	0.1	0.139	0.148	0.195	0.012	0.178	–0.028	0.02
Δ Eating disturbances	0.066	0.19	0.21	0.089	0.091	0.171	0.067	0.048
Δ Treatment burden	0.133	0.029	–0.053	–0.094	–0.073	0.019	0.158	0.043
Δ Health perception	0.325**	0.156	0.191	–0.049	–0.24	–0.054	0.137	0.247*
Δ Weight problems	0.545***	0.19	0.162	0.345**	–0.189	–0.125	0.340**	0.366**
Δ Respiratory symptoms	0.177	0.015	0.205	0.069	–0.112	–0.104	0.041	0.112
Δ Digestive symptoms	–0.04	–0.081	0.027	–0.012	–0.039	–0.066	0.056	–0.004

Data are Spearman rank correlation coefficients. A positive correlation indicates that a higher level of physical activity or fitness is associated with a better QoL.

Abbreviations: *MVPA* moderate–and–vigorous physical activity, *Wmax* maximal aerobic power, *VO2peak* peak oxygen uptake.

Significant correlation coefficients are highlighted by bold characters.

*– p < 0.05; **– p < 0.01; ***– p < 0.001.

### Relationship between HRQoL and physical activity or fitness

Cross-sectional and longitudinal relationships between HRQoL, physical activity and fitness are shown in Table 4. The HRQoL scale ‘Social role’ (i.e. a high value denotes little impairment in social role) was directly related to reported physical activity. Changes in reported physical activity over 6 months were directly related to changes in the ‘Role limitations’, ‘Feelings of embarrassment’, and ‘Weight problems’ scales. Belonging to the strength training group was associated with a lower HRQoL-scale ‘Vitality’ (data not shown). Objectively measured physical activity indices showed no significant associations with any HRQoL scale, neither in the cross-sectional nor in the longitudinal data analyses.

In the cross-sectional analyses, muscle power was directly associated with the HRQoL-scales ‘Physical functioning’, ‘Vitality’, ‘Role imitations’, and ‘Digestive symptoms’ while no associations were observed between changes in muscle power and changes in any HRQoL-scale in the longitudinal analyses. Aerobic power (Wmax) directly correlated with 4 HRQoL scales cross-sectionally and a change in aerobic power over 6 months was directly associated with ‘Vitality’, ‘Role limitations’ and ‘Weight problems’ scales. VO_2_peak even positively correlated with 7 of the 13 HRQoL scales cross-sectionally, including 5 disease-oriented scales. Furthermore, a change in VO_2_peak over 6 months directly correlated with the HRQoL-scales ‘Feelings of embarrassment’, ‘Health perception’ and ‘Weight problems’. The strength of the associations were moderate and ranged from r = 0.25-0.46.

## Discussion

In this study we found that aerobic fitness and reported physical activity were directly and moderately associated with HRQoL scales cross-sectionally and to a lesser extent also longitudinally in a sample of 76 patients with CF and mild to moderate disease. Established determinants of HRQoL such as FEV1 and BMI correlated with fewer scales. Based on scarce data in the literature looking at the relation between HRQoL and aerobic fitness and physical activity in CF, these findings are most important and relevant from a clinical perspective. The novel aspect of including longitudinal associations strengthens the findings.

HRQoL in our study was similar to that reported for patients with mild to moderate disease severity [5,11]. In contrast to the German [11] and American [5] validation studies of the CFQ-R, data of patients with an FEV1 below 35% predicted were not included in our analyses which reduced the range of FEV1 in our project (35-107% predicted) compared to the respective validation studies (Germany: 12-106% predicted; United States: 17-130% predicted) [5,11]. Possibly as a consequence of the smaller range in FEV1, surprisingly few significant associations were observed between HRQoL-scales and FEV1 in the current project [5,11]. Nevertheless, the power of our study was sufficient to detect clear and consistent positive associations between physical fitness and multiple HRQoL-scales.

### Relationship between HRQoL and physical activity

In contrast to our hypothesis, but in accordance to another comparable study in children with CF [12] no significant associations were observed between objectively measured physical activity and any HRQoL-scale. For reported physical activity, only one significant direct correlation was detected with the scale ‘Role limitations’ cross-sectionally (Table 4A). In a previous cross-sectional study, physical activity was found to correlate quite strongly with Quality of Well-being reported by patients with CF [8]. The discrepancy to our findings might reflect differences in the approach to determine HRQoL between the generic Quality of Well-being Scale and the multi-dimensional disease-specific CFQ-R. The Quality of Well-being Scale asks specifically whether or not the patient performs certain activities while the CFQ-R assesses the patient’s feelings and difficulties while performing activities.

Changes in reported physical activity, however, were positively associated with changes in three scales of the CFQ-R in the current study, namely ‘Role limitations’, ‘Feelings of embarrassment’, and ‘Weight problems’. This is important, as it shows that an increase in physical activity could increase HRQoL even after just 6 months. Other studies have also shown significant positive relationships between self-reported physical activity and HRQoL in healthy adults [6] or patients with chronic obstructive pulmonary disease [26].

While reported physical activity showed some direct association with HRQoL, objectively measured activity did not. This finding is difficult to interpret. Possibly, specific aspects of our study design have obscured a significant relationship: Accelerometry was performed within a given period of three weeks before or after assessment of HRQoL, while anthropometry, pulmonary function assessment, exercise testing, and completion of activity and quality of life questionnaires were all done on the same day. It is possible that physical activity assessed by accelerometry over a few days did not mirror physical activity a few weeks earlier or later which may have been confounded by short-term alterations in disease state or weather conditions [27]. Furthermore, the approach chosen to analyze accelerometer data including the selection of epoch times, definition of non-wear time or cutoffs for MVPA has been shown to influence the results [28] and may also have contributed to the attenuated relation between physical activity and HRQoL.

Furthermore, due to incomplete accelerometry data, sample size for the respective analyses and thus statistical power was lower than for other variables. Further studies are required to explore the associations between objectively measured physical activity and HRQoL in CF.

### Relationship between HRQoL and physical fitness

The main finding of the present study was that physical fitness, mainly aerobic fitness, was positively associated with HRQoL cross-sectionally and longitudinally. This observation is in line with the positive association between changes in aerobic fitness and changes in Quality of Well-being reported by others [29] and the direct associations between VO_2_peak and the HRQoL-scales ‘physical functioning’, ‘emotional functioning’, ‘social functioning’, and ‘treatment burden’ observed by Groenevelt et al. [12]. Furthermore, the significant and positive correlations between indicators of aerobic fitness and the disease-specific HRQoL-scales were as strong and sometimes stronger than the associations of these scales with more traditional measures of disease severity such as age, FEV1 or body composition. This finding underlines the importance of a good physical fitness for HRQoL in CF especially in disease-specific scales, and therefore the importance of physical fitness assessments in patients with CF. In addition, our findings strengthen the construct validity of the CFQ-R.

After adjusting for age and gender, the only other cross-sectional study testing the relation between physical activity, physical fitness, and HRQoL scales did not find any significant correlation [12]. This may be due to the much smaller sample size in their study group and the large age ranges as important confounders. Furthermore, the expression of the aerobic fitness per kg bodyweight rather than per predicted value may have “penalized” those who maintained body weight (leading to lower VO_2_peak per kg values) and “favorized” those who lost bodyweight (leading to higher VO_2_peak per kg values) – the opposite scenario from what would be expected from a clinical perspective. Indeed, when analyzing our data with a multiple regression model and adjusting for age, gender and nationality, direct relationships between VO_2_peak and 9 HRQoL-scales were observed (see Additional file 1).

At first sight it seems surprising that the HRQoL-scales ‘Physical functioning’ and ‘Respiratory symptoms’ were not responsive to changes in physical fitness. It is notable, however, that the actual data were taken from a training study and not from a longitudinal set of observational data. Thus, the patients included in this project were willing and felt generally capable of participating in regular exercise. This approach might have affected HRQoL perception related to changes in physical fitness. Moreover, several training studies have shown that physical conditioning may improve physical fitness while FEV1 remains relatively unaffected [12,30]. Thus, although there was a significant direct correlation between peak oxygen uptake and the ‘Respiratory symptom’ scale at baseline, the subjective and objective respiratory situation in many patients might not have changed enough to be perceived positively despite improvements in aerobic fitness.

One limitation of the current project is the relatively small number of patients in the five treatment conditions that limits statistical power in detecting effects of the different treatment regimens on HRQoL. Nevertheless, supervised strength training three times per week over six months was associated with significant decreases in the HRQoL-scale ‘Vitality’, i.e. less vitality. It is possible that the supervised strength training might have been too strenuous to maintain or even improve ‘Vitality’ despite improvements in aerobic fitness and pulmonary function [14]. A decrease in feelings of energy and an increase in perceived fatigue were observed in competitive swimmers progressively increasing their training volume [31], which supports our “overload” hypothesis. In contrast to our findings, others did not observe a negative effect of a presumably less strenuous partially supervised strength training program on Quality of Well-being [32] which presumes that the negative effects on some HRQoL-scales in our study were rather a consequence of the intensity than of the strength training per se.

The discrepancies in time spent active between the subjective and objective assessments might be perceived as a further limitation of the study. However, this discrepancy is possibly related to the already mentioned different time frame that the two measurement tools covered and the way accelerometer data were analyzed that may at least in part have attenuated associations with HRQoL. In addition, reporting bias based on social desirability and social approval may have occurred [33].

Furthermore, as this study was a training study, it cannot be completely excluded that the interaction of the training supervisors with the participants may have affected some of the associations between the fitness, activity and HRQoL variables. Nevertheless, the fact that there was merely no difference in changes of HRQoL among groups suggests that this influence was minor. Finally, there is a possibility of shared variance amongst a number of the predictor variables in the regression model explaining HR-QoL that could be responsible for a reduced association between variables. Yet, this potential multicollinearity does not reduce the predictive power or reliability of the model as a whole but rather affects calculations regarding individual predictors.

Since physical conditioning programs in CF have been shown to improve pulmonary function, physical fitness and HRQoL in concert [13,14,29,30,34], patients should be encouraged to engage in regular at least moderate physical activities efficient to improve physical fitness even though we could only show beneficial effects of increased physical activity on some HRQoL-scales. To maintain or even improve ‘Vitality’, too strenuous training sessions without sufficient recovery periods such as unfamiliar strength training exercises three times per week need to be avoided in order to not compromise HRQoL.

## Conclusions

Health-related quality of life was directly associated with physical fitness cross-sectionally and longitudinally and, to a lesser extent, with reported physical activity, BMI and FEV1. These findings underline the importance of high physical fitness as a contributor to health-related quality of life in patients with cystic fibrosis and provide an additional rationale for exercise testing in this population.

## Abbreviations

CF: Cystic fibrosis; CFQ-R: Revised cystic fibrosis questionnaire; HRQoL: Health-related quality of life; MVPA: Moderate-and-vigorous physical activity; VO2peak: Peak oxygen uptake; Wmax: Maximal aerobic power.

## Competing interests

The authors declare that they have no competing interests.

## Authors’ contributions

HH and SuK developed the concept and the design of the two twin studies with important input from MB. HH, StK, SJ, MB, AH, TS, HP, and SuK collected the data. HH, KS, KR, CS, and SuK performed the analyses and interpreted the data. HH drafted the manuscript with all authors critically revising it for important intellectual content. All authors have given final approval of the version to be published.

## Pre-publication history

The pre-publication history for this paper can be accessed here:

http://www.biomedcentral.com/1471-2466/14/26/prepub

## Supplementary Material

Additional file 1 Relationships of baseline (A) and changes over 6 months (B) between quality of life scales and BMI, FEV1, physical activity measures, muscle power, and aerobic capacity by mulitple linear regression analysis after adjusting for age, gender, and nationality in the cross-sectional analyses and for age, gender, nationality, training vs no training, strength training vs other training modalities in the longitudinal analyses. Click here for file

## References

[B1] Goss CH Quittner AL Patient-reported outcomes in cystic fibrosis Proc Am Thorac Soc 2007 4 378 386 10.1513/pats.200703-039BR 17652505 PMC2647603

[B2] Abbott J Hart A Measuring and reporting quality of life outcomes in clinical trials in cystic fibrosis: a critical review Health Qual Life Outcomes 2005 3 19 10.1186/1477-7525-3-19 15790407 PMC1079915

[B3] Abbott J Hart A Morton AM Dey P Cornway SP Webb AK Can health-related quality of life predict survival in adults with cystic fibrosis? Am J Respir Crit Care Med 2009 179 54 58 10.1164/rccm.200802-220OC 18948427

[B4] Gee L Abbott J Conway SP Etherington C Webb AK Development of a disease specific health related quality of life measure for adults and adolescents with cystic fibrosis Thorax 2000 55 946 954 10.1136/thorax.55.11.946 11050265 PMC1745639

[B5] Quittner AL Buu A Messer MA Modi AC Watrous M Development and validation of the cystic fibrosis questionnaire in the United States - a health-related quality-of-life measure for cystic fibrosis Chest 2005 128 2347 2354 10.1378/chest.128.4.2347 16236893

[B6] Bize R Johnson JA Plotnikoff RC Physical activity level and health-related quality of life in the general adult population: a systematic review Prev Med 2007 45 401 415 10.1016/j.ypmed.2007.07.017 17707498

[B7] Sloan RA Sawada SS Martin CK Church T Blair SN Associations between cardiorespiratory fitness and health-related quality of life Health Qual Life Outcomes 2009 7 47 10.1186/1477-7525-7-47 19476640 PMC2695434

[B8] Selvadurai HC Blimkie CJ Cooper PJ Mellis CM Van Asperen PP Gender differences in habitual activity in children with cystic fibrosis Arch Dis Child 2004 89 928 933 10.1136/adc.2003.034249 15383436 PMC1719659

[B9] Orenstein DM Nixon PA Ross EA Kaplan RM The quality of well-being in cystic fibrosis Chest 1989 95 344 347 10.1378/chest.95.2.344 2914486

[B10] Congleton J Hodson ME Duncan-Skingle F Do Nottingham Health Profile scores change over time in cystic fibrosis? Respir Med 1998 92 268 272 10.1016/S0954-6111(98)90107-X 9616524

[B11] Wenninger K Aussage P Wahn U Staab D and the Germany CFQ study group The revised German Cystic Fibrosis Questionnaire: validation of a disease-specific health-related quality of life instrument Qual Life Res 2003 12 77 85 10.1023/A:1022011704399 12625520

[B12] Groeneveld IF Sosa ES Pérez M Fiuza-Luces C Gonzalez-Saiz L Gallardo C López-Mojares LM Ruiz JR Lucia A Health-related quality of life of Spanish children with cystic fibrosis Qual Life Res 2012 21 1837 1845 10.1007/s11136-011-0100-8 22219170 PMC3496548

[B13] Hebestreit H Kieser S Junge S Ballmann M Hebestreit A Schenk T Posselt HG Kriemler S Long-term effects of a partially supervised conditioning programme in cystic fibrosis Eur Respir J 2010 35 578 583 10.1183/09031936.00062409 19643946

[B14] Kriemler S Kieser S Junge S Ballmann M Hebestreit A Schindler C Stüssi C Hebestreit H Effect of supervised training on FEV1 in cystic fibrosis J Cyst Fibros 2013 12 714 720 10.1016/j.jcf.2013.03.003 23588193

[B15] Slaughter MH Lohman TG Boileau RA Horswill CA Stillman RJ Van Loan MD Bemben DA Skinfold equations for estimation of body fatness in children and youth Hum Biol 1988 60 709 723 3224965

[B16] Durnin JVGA Womersley J Body fat assessed from total body density and its estimation from skinfold thickness: measurements on 481 men and women aged from 16 to 72 years Br J Nutr 1974 32 77 97 10.1079/BJN19740060 4843734

[B17] Sherrill DL Lebowitz MD Knudson RJ Burrows B Continuous longitudinal regression equations for pulmonary function measures Eur Respir J 1992 5 452 462 1563504

[B18] Inbar O Bar-Or O Skinner JS The Wingate Anaerobic Test 1996 Champaign, IL: Human Kinetics

[B19] Williams CA Doré E Alban J van Praagh E Short-term power output in 9-year-old children: typical error between ergometers and protocols Ped Exerc Sci 2003 15 302 312

[B20] Godfrey S Davies CTM Wozniak E Barnes CA Cardio-respiratory response to exercise in normal children Clin Sci 1971 40 419 431 5556096 10.1042/cs0400419

[B21] Orenstein DM Rowland TW Assessment of exercise pulmonary function Pediatric Laboratory Exercise Testing. Clinical Guideline.s 1993 Champaign, IL: Human Kinetics 141 163

[B22] Sallis JF Haskell WL Wood PD Fortmann SP Rogers T Blair SN Paffenbarger RS Jr Physical activity assessment methodology in the five-city project Am J Epidemiol 1985 121 91 106 3964995 10.1093/oxfordjournals.aje.a113987

[B23] Sallis JF Buono MJ Roby JJ Micale FG Nelson JA Seven-day recall and other physical activity self-reports in children and adolescents Med Sci Sports Exerc 1993 25 99 108 10.1249/00005768-199301000-00014 8423762

[B24] Ruf KC Fehn S Bachmann M Moeller A Roth K Kriemler S Hebestreit H Validation of activity questionnaires in patients with cystic fibrosis by accelerometry and cycle ergometry BMC Med Res Methodol 2012 12 43 10.1186/1471-2288-12-43 22471343 PMC3359253

[B25] Hebestreit H Kieser S Rüdiger S Schenk T Junge S Hebestreit A Ballmann M Posselt HG Kriemler S Physical activity is independently related to aerobic capacity in cystic fibrosis Eur Resp J 2006 28 734 739 10.1183/09031936.06.00128605 16807261

[B26] McGlone S Venn A Walters EH Physical activity, spirometry and quality-of-life in chronic obstructive pulmonary disease COPD 2006 3 83 88 10.1080/15412550600651263 17175670

[B27] Chan CB Ryan DA Tudor-Locke C Relationship between objective measures of physical activity and weather: a longitudinal study Int J Behav Nutr Phys Act 2006 3 21 10.1186/1479-5868-3-21 16893452 PMC1557535

[B28] Corder K Ekelund U Steele RM Wareham NJ Brage S Assessment of physical activity in youth J Appl Physiol 2008 105 977 987 10.1152/japplphysiol.00094.2008 18635884

[B29] Selvadurai HC Blimkie CJ Meyers N Mellis CM Cooper PJ VanAsperen PP Randomized controlled study of in-hospital exercise training programs in children with cystic fibrosis Pediatr Pulmonol 2002 33 194 200 10.1002/ppul.10015 11836799

[B30] Orenstein DM Franklin BA Doerchuk CF Hellerstein HK Germann KJ Horowitz JG Stern RC Exercise conditioning and cardiopulmonary fitness in cystic fibrosis The effects of a three-month supervised running program. Chest 1981 80 392 398 10.1378/chest.80.4.392 7023863

[B31] Raglin JS Morgan WP O´Connor PJ: **Changes in mood states during training in female and male college swimmers** Int J Sports Med 1991 12 585 589 10.1055/s-2007-1024739 1797703

[B32] Orenstein DM Hovell MF Mulvihill M Keating KK Hofstetter R Kelsey S Morris K Nixon PA Strength vs aerobic training in children with cystic fibrosis Chest 2004 126 1204 1214 10.1378/chest.126.4.1204 15486384

[B33] Adams SA Matthews CE Ebbeling CB Moore CG Cunningham JE Fulton J Hebert JR The effect of social desirability and social approval on self-reports of physical activity Am J Epidemiol 2005 161 389 398 10.1093/aje/kwi054 15692083 PMC2958515

[B34] Schneiderman-Walker J Pollock SL Corey M Wilkes DD Canny GJ Pedder L Reisman JJ A randomized controlled trial of a three year home exercise program in cystic fibrosis J Pediatr 2000 136 304 310 10.1067/mpd.2000.103408 10700685

