# Supplementary material for: Quality of life is associated with physical activity and fitness in cystic fibrosis
Source: BMC Pulm Med. 2014 Feb 27;14:26. doi: 10.1186/1471-2466-14-26 (PMC3942299; doi:10.1186/1471-2466-14-26)
Supplement: Additional file 1 — Relationships of baseline (A) and changes over 6 months (B) between quality of life scales and BMI, FEV1, physical activity measures, muscle power, and aerobic capacity by mulitple linear regression analysis after adjusting for age, gender, and nationality in the cross-sectional analyses and for age, gender, nationality, training vs no training, strength training vs other training modalities in the longitudinal analyses. [file 1471-2466-14-26-S1.docx]

| **Additional file 1** Relationships of baseline (A) and changes over 6 months (B) between quality of life scales and BMI, FEV1, physical activity measures, muscle power, and aerobic capacity by regression analysis | | | | | | | | |
| --- | --- | --- | --- | --- | --- | --- | --- | --- |
| A. baseline |  |  |  |  |  |  |  |  |
|  | BMI | Body fat | FEV1 | Reported activity | Measured MVPA | Muscle power | Wmax | VO_2_peak |
| Physical functioning | 1.11  (-0.36;2.59) | 0.17  (-0.53;0.88) | **0.23***  **(0.02;0.43)** | 0.24  (-0.16;0.64) | 0.07  (-0.69;0.83) | 4.53  (-0.12;9.19) | **0.24****  **(0.07;0.41)** | **0.36****  **(0.11;0.61)** |
| Vitality | 0.53  (-0.93;1.99) | 0.30  (-0.45;1.06) | -0.03  (-0.26;0.20) | **0.36***  **(0.01;0.72)** | -0.30  (-1.15;0.55) | **4.14***  **(0.43;7.85)** | 0.15  (-0.05;0.36) | **0.27***  **(0.01;0.53)** |
| Emotional state | 0.72  (-0.74;2.19) | 0.21  (-0.58;1.00) | -0.03  (-0.21;0.16) | 0.20  (-0.10;0.50) | 0.14  (-0.62;0.90) | 1.03  (-2.52;4.59) | 0.14  (-0.01;0.29) | **0.23***  **(0.01;0.45)** |
| Social limitations | 0.40  (-2.05;2.85) | 0.41  (-0.58;1.41) | -0.12  (-0.40;0.15) | 0.35  (-0.30;1.00) | -0.63  (-1.59;0.33) | -1.98  (-7.47;3.50) | 0.10  (-0.14;0.35) | 0.13  (-0.23;0.48) |
| Role limitations | 0.07  (-1.34;1.47) | 0.24  (-0.52;1.00) | 0.14  (-0.01;0.30) | 0.29  (-0.05;0.63) | 0.55  (-0.07;1.18) | 1.02  (-1.93;3.98) | **0.16***  **(0.03;0.29)** | **0.27****  **(0.09;0.45)** |
| Feelings of embarrassment | -0.01  (-2.52;2.50) | 0.62  (-0.72;1.96) | 0.11  (-0.16:0.37) | 0.46  (-0.25;1.17) | 0.36  (-0.97;1.69) | -1.21  (-7.29;4.88) | 0.07  (-0.19;0.33) | 0.22  (-0.13;0.57) |
| Body image | **3.83*****  **(1.76;5.90)** | **1.41***  **(0.34;2.49)** | **0.32***  **(0.07;0.56)** | 0.43  (-0.07;0.93) | 0.48  (-0.71;1.67) | -4.39  (-10.20;1.42) | 0.20  (-0.07;0.47) | 0.33  (-0.02;0.68) |
| Eating disturbances | **2.97****  **(1.16;4.77)** | 0.50  (-0.50;1.50) | 0.11  (-0.14;0.35) | 0.01  (-0.55;0.57) | 0.28  (-0.90;1.46) | -0.36  (-5.49;4.77) | 0.20  (-0.01;0.40) | **0.29***  **(0.01;0.57)** |
| Treatment burden | 1.01  (-0.86;2.88) | 0.46  (-0.55;1.47) | -0.04  (-0.28;0.21) | -0.04  (-0.55;0.47) | -0.35  (-1.32;0.61) | 2.03  (-1.91;5.98) | 0.15  (-0.09;0.39) | 0.24  (-0.07;0.55) |
| Health perception | **2.06***  **(0.39;3.72)** | 0.51  (-0.35;1.37) | **0.25***  **(0.01;0.50)** | 0.23  (-0.22;0.67) | 0.76  (-0.26;1.77) | -1.04  (-6.12;4.03) | **0.31****  **(0.08;0.53)** | **0.53*****  **(0.23;0.82)** |
| Weight problems | **4.50*****  **(2.02;7.00)** | 0.28  (-1.00;1.56) | 0.20  (-0.11;0.51) | -0.22  (-1.16;0.72) | 0.60  (-1.02;2.22) | -0.35  (-7.41;6.70) | **0.47****  **(0.20;0.75)** | **0.75*****  **(0.43;1.07)** |
| Respiratory symptoms | 0.70  (-0.51;1.92) | 0.29  (-0.40;0.99) | 0.17  (-0.01;0.35) | 0.26  (-0.00;0.52) | 0.27  (-0.29;0.84) | 1.12  (-2.09;4.33) | **0.18***  **(0.03;0.34)** | **0.35****  **(0.15;0.56)** |
| Digestive symptoms | 0.72  (-0.86;2.30) | -0.19  (-1.12;0.74) | 0.02  (-0.18;0.22) | 0.23  (-0.20;0.66) | 0.68  (-0.19;1.55) | **4.87***  **(0.89;8.76)** | **0.25****  **(0.09;0.42)** | **0.39*****  **(0.18;0.60)** |
| B. changes over 6 months |  |  |  |  |  |  |  |  |
|  | Δ BMI | Δ Body fat | Δ FEV1 | Δ Reported activity | Δ Measured MVPA | Δ Muscle power | Δ Wmax | Δ VO_2_peak |
| Δ Physical functioning | 0.70  (-1.90;3.30) | 0.36  (-0.88;1.60) | 0.11  (-0.09;0.31) | 0.15  (-0.13;0.43) | -0.37  (-1.27;0.52) | 2.09  (-2.45;6.63) | 0.12  (-0.07;0.31) | 0.16  (-0.04;0.36) |
| Δ Vitality^$^ | **4.07***  **(0.73;7.41)** | 1.53  (-0.08;3.14) | 0.06  (-0.47;0.60) | **0.45***  **(0.04**;**0.87)** | -1.14  (-2.41;0.13) | 4.54  (-0.37;9.45) | **0.24***  **(0.01**;**0.47)** | 0.21  (-0.21;0.64) |
| Δ Emotional state | **5.41***  **(0.21;10.60)** | 1.65  (-0.67;3.98) | 0.14  (-0.30;0.58) | 0.04  (-0.28;0.36) | -0.41  (-1.45;0.62) | 3.71  (-2.61;10.03) | 0.07  (-0.18;0.31) | 0.10  (-0.28;0.48) |
| Δ Social limitations | 0.99  (-3.82;5.81) | 0.54  (-1.45;2.53) | 0.13  (-0.38;0.65) | 0.05  (-0.41;0.50) | -0.47  (-1.44;0.51) | -0.16  (-7.71;7.39) | 0.16  (-0.17;0.48) | **0.36***  **(0.01;0.72)** |
| Δ Role limitations | 3.84  (-1.21;8.89) | 0.09  (-1.89;2.07) | 0.03  (-0.33:0.40) | 0.47  (-0.07;1.00) | -0.05  (-1.85;1.75) | 3.36  (-1.81;8.52) | 0.23  (-0.12;0.58) | 0.15  (-0.30;0.60) |
| Δ Feelings of   embarrassment | 2.90  (-2.59;8.40) | 1.87  (-0.85;4.59) | 0.30  (-0.40;0.99) | **0.77***  **(0.11;1.43)** | -0.20  (-1.27;0.87) | -1.93  (-6.73;2.88) | 0.28  (-0.06;0.61) | 0.29  (-0.12;0.70) |
| Δ Body image | 3.73  (-0.99;8.46) | 1.73  (-0.34;3.80) | 0.37  (-0.07;0.81) | 0.35  (-0.15;0.85) | 0.31  (-0.83;1.45) | 3.25  (-1.78;8.29) | 0.03  (-0.37;0.43) | 0.09  (-0.37;0.54) |
| Δ Eating disturbances | 3.13  (-1.70;7.96) | 1.03  (-0.55;2.61) | 0.22  (-0.22;0.66) | -0.15  (-0.52;0.23) | 0.5  (-0.60;1.60) | 3.73  (-0.04;7.49) | 0.09  (-0.28:0.45) | 0.08  (-0.33;0.49) |
| Δ Treatment burden | 2.26  (-3.00;7.53) | 0.28  (-2.08;2.65) | 0.10  (-0.40;0.60) | -0.19  (-0.80;0.42) | -0.05  (-1.02;0.93) | -0.99  (-7.63;5.65) | 0.32  (-0.03;0.68) | 0.19  (-0.31;0.69) |
| Δ Health perception | **7.08*****  **(3.45;10.71)** | 2.08  (-0.16;4.31) | 0.37  (-0.07;0.81) | -0.13  (-0.60;0.34) | -0.38  (-1.73;0.96) | -0.19  (-7.78;7.40) | 0.11  (-0.19;0.40) | **0.42***  **(0.05;0.80)** |
| Δ Weight problems | **20.86*****  **(14.51;27.21)** | 4.87  (-0.26;10.01) | 0.31  (-0.65;1.27) | 0.76  (-0.17;1.69) | -0.76  (-3.61;2.08) | -1.13  (-13.16;10.89) | **0.93****  **(0.24;1.62)** | **1.10***  **(0.13;2.07)** |
| Δ Respiratory symptoms | 1.84  (-3.34;7.06) | -0.48  (-2.38;1.42) | **0.58***  **(0.10;1.07)** | 0.18  (-0.23;0.59) | -0.30  (-1.18;0.57) | 1.13  (-5.41;7.67) | -0.03  (-0.37;0.31) | 0.14  (-0.16;0.44) |
| Δ Digestive symptoms | 0.14  (-4.05;4.33) | 0.90  (-3.00;1.19) | 0.09  (-0.33;0.51) | -0.00  (-0.54;0.53) | -0.55  (-1.66;0.56) | -1.38  (-7.17;4.41) | -0.02  (-0.42;0.38) | -0.08  (-0.52;0.36) |
| Data are β-coefficients of a multiple linear regression model with bootstrapping after adjusting for age, gender, and nationality (A. cross-sectional analyses) and for age, gender, nationality, training vs no training, strength training vs other training modalities (B. longitudinal analyses). A positive coefficient indicates that a higher level of BMI, body fat, FEV1, physical activity or fitness is associated with a better HR-QoL. Abbreviations: MVPA – moderate-and-vigorous physical activity, Wmax – maximal aerobic power, VO_2_peak – peak oxygen uptake. $ - significant negative effect of weight training on Δ Vitality compared to no weight training. Significant correlation coefficients are highlighted by bold characters. * - p<0.05; ** - p<0.01; *** - p<0.001 | | | | | | | | |
